# Prevalence and Correlates of Obesity in Schoolchildren from the City of Bursa,Turkey

**DOI:** 10.4008/jcrpe.v1i2.15

**Published:** 2008-11-04

**Authors:** Halil Sağlam, Ömer Tarım

**Affiliations:** 1 Uludağ University Faculty of Medicine, Department of Pediatrics, Division of Pediatric Endocrinology, Görükle, Bursa, Turkey; +90−224−442 81 43+90−224−442 81 43drhalil@uludag.edu.trUludağ University Medical Faculty, Department of Pediatrics, Division of Pediatric Endocrinology, Görükle, Bursa, Turkey

**Keywords:** childhood, obesity, prevalence, etiology

## Abstract

**Background and objectives**: The prevalence of childhood obesity has been dramatically increasing worldwide. This study was performed to examine the prevalence and etiological factors of obesity in children aged 6−12 years and to investigate the relative contribution of exogenous factors with respect to sociodemographic data.

**Methods**: A total of 5368 children aged 6−12 years in eight urban elementary schools located in Bursa, the fourth largest city of Turkey, were included in this cross−sectional study. A dietary record for three days and a questionnaire for the assessment of socio−economic and demographic parameters were completed by the parents at home. The height and weight of the children were measured and relative weight and body mass index (BMI) were calculated.

**Results**: The prevalence of overweight, obesity and severe obesity according to BMI were 12.4%, 7.8% and 2.2%, respectively. The female/male ratio among obese children was 1.24. Eighty percent of obese children had one or both parents obese. Age, gender, presence of obesity in parents, higher educational level of the parents, consumption of soft beverages such as soda and juice, physical activity level and higher income of the family were found as the contributing factors to obesity.

**Conclusion**: The prevalence of obesity is increasing in Turkey in parallel to the trend in many countries. There are consistent and predictable sociodemographic parameters that are associated with or may impose a risk factor for obesity. Identification of these risk factors will provide areas to target in the prevention and management of this common problem.

**Conflict of interest:**None declared.

## INTRODUCTION

Obesity is defined as increase in body weight due to accumulation of fat.(1) Many criteria and methods of measurement have been developed to diagnose and assess the severity of obesity. Among these, body mass index (BMI) defined for age and sex is the most popular and probably the most practical and accurate way of diagnosing and monitoring obesity.(1, 2, 3)

The prevalence of obesity in childhood has increased all over the world over the past decade.(4, 5, 6, 7, 8, 9, 10, 11, 12, 13, 14, 15, 16, 17)

The recent advances in technology have dramatically changed the life−style of children in many countries. Electronic amusements have replaced outside activities and motor vehicles and telephones have reduced the distances to walk. “Fast foods” have been the food of choice not only because of time constraints, but also because of the competitive advertisements of the fast food companies. Larger portions for lower prices have been the basic theme of this competition. These changes in life−style are also happening in Turkey and, as the country becomes more westernized, childhood obesity is expected to increase more dramatically.

Obesity leads to important morbidities such as hypertension, type 2 diabetes, hyperlipidemia, hyperuricemia, orthopedic, and psychosocial complications and it is directly related to all mortalities.(18, 19) It is well known to patients and physicians that management of obesity is a difficult and frustrating endeavour. Therefore, prevention seems to be a better and more successful strategy to handle this epidemic. Identification of associated risk factors will provide insight to develop methods of prevention and intervention.

In this cross−sectional, epidemiologic study, we aimed to determine the prevalence of obesity in schoolchildren and investigate the associated sociodemographic factors that may impose a risk for excess weight gain. Validity of different parameters used in assessment of obesity was also investigated.

## METHODS

A total of 5368 children aged 6−12 years in eight elementary schools in Bursa, which is the fourth largest city of Turkey with a population of over 2 million, were screened cross−sectionally for obesity by using a stratified sampling method. To get an equal distribution of children by socio−economic strata, the schools were selected from three district municipalities (DM) [Nilüfer (DM1) with a per capita income of about 8000 USD, Osmangazi (DM2) with a per capita income of about 4500 USD, and Yıldırım (DM3) with a per capita income of about 1500 USD according to State Statistics Institute of the city. The numbers of children randomly enrolled in DM1, DM2, and DM3 were 1788 (32.4%), 1832 (33.2%), and 1902 (34.4%), respectively. Children were enrolled from 8 randomly chosen governmental schools (3 out of 24 schools from DM1, 3 out of 27 schools from DM2, and 2 from 17 schools from DM3). All children from the first to fifth grades in each school who attended to school on the day of the examination and who returned the previously distributed questionnaire were included in the study. Those children who rejected participation were excluded. Children were informed about the study by the school administration and by one of the researchers (HS).

Research approval was obtained from the Ethical Committee of the Uludağ University. Written consent was obtained from the parents of the children through the school administration for anthropometric measurements and for data collection by questionnaires distributed to the children.

A dietary record for three days was requested to be recorded and provided on the day of anthropometric measurements with the questionnaire. Three days prior to the anthropometric measurements, all the classes were visited; children were informed about the study and the questionnaire. The questionnaire was designed to provide information on socioeconomic and demographic parameters and included questions on physical activity, food habits, occupation of the parents, socio−economic status of the family, birth date and birth weight of the child. The questionnaires were distributed by the school administration to be filled by the parents at home. The children were advised to take home the questionnaire to their parents to fill in the details. Parents were requested to have their heights and weights measured. The dietary records and the filled questionnaires were collected when the school was visited by the pediatricians for anthropometric measurements.

The socio−economical status (SES) of the family was graded as low, moderate or high according to our scoring system including the items of total family income, income per person in the family, owning a house, a car, a computer and the room numbers of the house (a total of 10 scores). Physical activity behaviour was assessed by asking how many times per week the subjects “played or exercised enough to make them sweat and breathe hard”. These activities did not exclude school−related involvements such as physical education. Children who reported 3 or more sessions per week, each of which lasted at least one hour of physical activity, were classified as active. Those reporting fewer than 3 sessions were in the low−activity group. The questionnaire also included a question asking the number of hours the child had spent watching television the day preceding the examination. Respondents were grouped into 3 or more hours or fewer than 3 hours of watching TV per day. Diet score was calculated by adding the frequency per week of intermediate food items such as ice−creams, sweets, soft drinks, chocolates, fried foods, fast foods and eating out. This was validated against the daily consumption of calories calculated by using a 3− day diet record method. In the diet record form, the children and their parents were asked to record everything the child had eaten and drunk during 3 days before the examination day. Children were divided as those consuming intermediate food items more than 3 times per week and those consuming three times or less per week. Questionnaire responses and diet records were compared and discordant responses were excluded from the analysis.

In children whose questionnaire were returned completed, all height and weight measurements were performed and recorded by the same two paediatricians (HS, who is the main researcher and IE, who is a resident) using a portable stadiometer equipped with a mobile measuring stick perpendicular to the vertical line for height measurements. Weight was measured by the same portable device to the nearest 0.1 kg. The schoolchildren were measured while wearing street clothes (excluding sweaters, jackets, belts, and shoes). Two measurements for weight and height were performed for each child and the mean of those two measurements was used in all analyses. The measurement device was calibrated every day with a standard device at the university hospital.

The diagnostic criteria that were used for the evaluation of obesity included relative weight (RW) or percentage expected weight (PEW), BMI, and relative BMI. We aimed to compare these parameters in detecting the prevalence of obesity. RW or PEW was defined as actual weight divided by ideal (50th percentile) weight for height. A RW ratio between 110% and 120% was classified as overweight, a ratio between 120% to 140% as obesity and a ratio above 140% was considered as morbid obesity. BMI was defined as weight in kilograms divided by height in square meters (kg/m^2^). Relative BMI (RBMI) was defined as actual BMI divided by ideal (50^th^ percentile) BMI for age and sex. Again, a RBMI ratio between 110% and 120% was classified as overweight, between 120% and 140% as obesity and above 140% as morbid obesity. A BMI between 85^th^ to 95^th^ percentile for age and sex was classified as overweight, between 95^th^ to 99^th^ percentile as obesity and over 99^th^ percentile as morbid obesity.

All measured values were compared to CDC (Center for Disease Control) anthropometric reference dataset with the software called Growth, version 1.4 (last update of the growth data was May 30, 2000). Z−scores of weight, height, and BMI were all calculated by using this same software and were used in statistical analysis.

Statistical evaluation was performed with the aid of the computer program SPSS for Windows Version 10.1. Unpaired Student’s t−test, chi−square analysis, and logistic regression analysis were used for comparisons. The level of significance was assumed to be α=0.05.

## RESULTS

The completed questionnaire results of 5368 children were evaluated. Among these children, 2559 (47.7%) were girls and 2809 (52.3%) were boys. The overall response rate for the questionnaire was 97.2% (5368 out of 5522) and for the diet recording 78.3% (4326 out of 5522).

The prevalence of overweight was found to be 17.8% (19.6% for girls and 16.2% for boys), obesity 12.9% (15.9% for girls and 10.1% for boys) and morbid obesity 3.4% by the criterion of RW. When BMI was used as the diagnostic criterion, the prevalence of overweight, obesity and morbid obesity was calculated to be 12.4%, 7.8% and 2.2%, respectively and similar figures were calculated with the RBMI criterion (Table 1). Here, overweight children did not include obese and morbid obese children. Similarly obese children did not include morbid obese children (i.e., they are separate figures). The slight differences in these figures did not reach statistical significance level (p>0.05). For this reason, only BMI criterion was used when analyzing the data. The distribution of overweight and obesity according to age groups is given in Table 2. It is clearly seen that the prevalence tended to increase in parallel to the age.

Among the obese children, 55.3% were females (female/male ratio was 1.24). The difference between girls and boys with respect to obesity prevalence was not significant (p>0.05).

When SES scoring was considered, the Nilüfer district had the highest score (7.1±0.3), Yıldırım had the lowest score (4.1±0.2), and Osmangazi was between them (5.7±0.2). The differences between SES scores were statistically significant (p<0.05). The prevalence of overweight, obesity and morbid obesity significantly differed between the district municipalities (Table 3). As seen in this table, Nilüfer with the highest SES score had the highest overweight, obesity and morbid obesity rates. On the other hand, Yıldırım had the lowest rates.

When the parents of the obese children were studied, 66.2% of the mothers and 61.4% of the fathers had a BMI above 25 (Table 4). It was also found that 67.3% of the mothers and 60.2% of the fathers had emigrated from other parts of Turkey.

The sociodemographic factors that were associated with obesity included number of meals consumed per day, frequency of soda and juice consumption, parental age, BMI and education level of the parents, total and per person income of the family, physical activity, and the frequency of use of the elevator (Table 5). Children whose fathers and/or mothers were overweight or obese, whose fathers and or mothers had higher education, whose fathers and mothers were older, who consumed more than 3 meals per day, who consumed soda and juices more than 3 times per week, who participated in sport activities less than 3 times per week, who used elevator in everyday life, and whose socioeconomic status was higher were under greater risk of obesity.

Duration of breast feeding, introduction time of formulas, duration of formula feeding, transition age to additional foods were not correlated with relative weight, BMI, or RBMI. Likewise, daily TV hours and the number of people living in the house did not show any correlation with the indices of obesity.

**Table 1:**
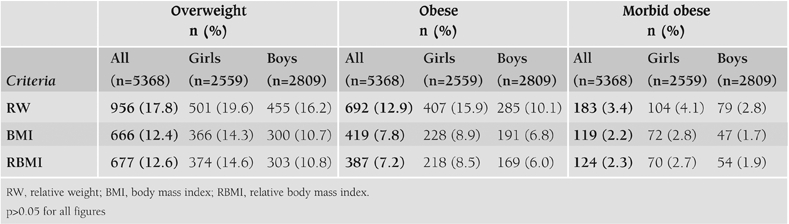
Number (%) of children classified as overweight, obese and morbid obese according to RW, BMI and RBMI criteria.

**Table 2:**
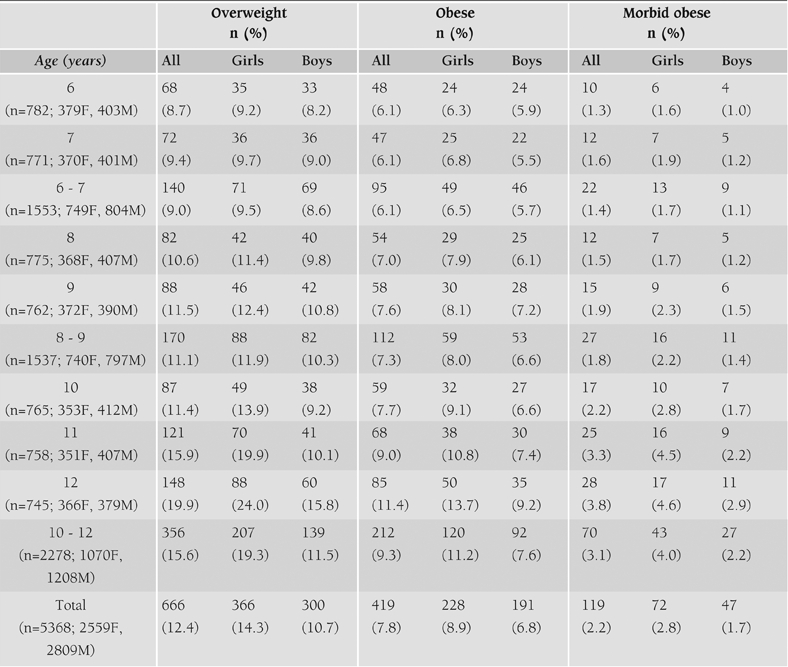
Distribution of overweight, obese, morbid obese girls and boys according to their ages.

**Table 3:**
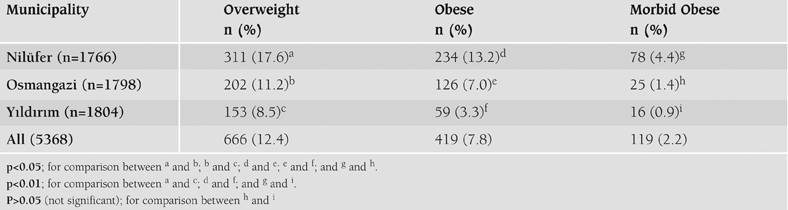
Distribution of overweight, obese and morbid obese children among district municipalities (according to BMI values).

**Table 4:**
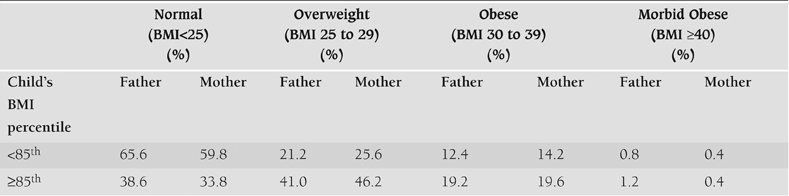
BMI distribution of the parents of children with a BMI percentile of <85^th^ or ≥85th.

**Table 5:**
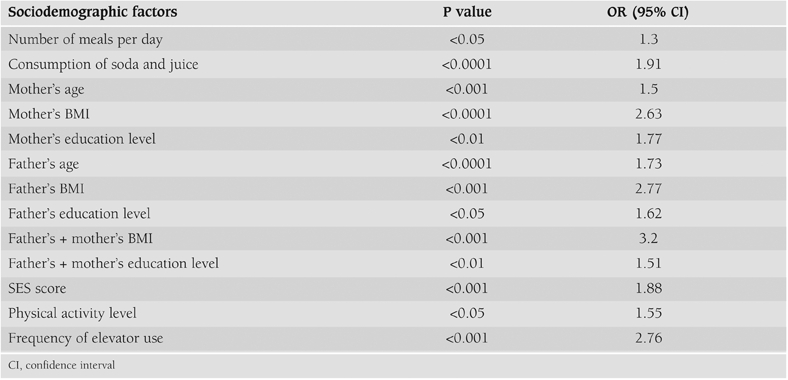
Correlation of sociodemographic factors with BMI.

## DISCUSSION

As demonstrated in the Bogalusa Heart Study, the prevalence of obesity doubled from 1973 to 1994.(8) The weight gain over this period was 3.4 kg for the 5−14−age−group and 5.7 kg for the 15−17−age−group. A similar trend has been reported from other countries.

Different methods including RW, BMI and RBMI have been used to evaluate obesity in children.(1, 2, 4, 8, 9) We showed that these three criteria produced similar figures in the evaluation of obesity in children. So, for the standardization of epidemiological studies, only one method and preferably BMI which is the most commonly used parameter should be used in all studies.

We think that the population we studied reflects the average of the country, because the mean SES of the families was representative of the middle class. In addition, Bursa is one of the cities that receives rural to urban emigration from all over the country; 67.3% of the mothers and 60.2% of the fathers in this study had moved from other parts of Turkey, as mentioned before.

Previous estimations of obesity prevalence in our country ranged between 2.6% and 6%. For example, the prevalence in adolescents was found 4.7% in Ankara in 1992.(20) Our results show that the problem has more than doubled over a time period of eleven years. Some recent similar studies also supported our results.(21, 22) It is even more worrisome if we take into account the fact that our study group consisted of pre−adolescents. There is considerable evidence that obese children become obese adolescents, most of whom eventually become obese adults.(14, 18, 23, 24)

The most important sociodemographic factors that seemed to be associated with obesity were consumption of soda and juice, parental age, BMI, education level of the parents, total and per person income of the family, physical activity, and the frequency of use of the elevator. Similar risk factors have been also demonstrated by other studies.(25, 26, 27, 28, 29, 30, 31, 32, 33, 34, 35) In the present study, as the number of meals consumed per day increased, so did the obesity prevalence, but this increase did not reach statistical significance (p=0.055).

As seen from our results, in Turkey, obesity is largely a problem of higher socio−economic status people. Children living in a rich family or area have an increased risk of obesity. Even the higher educational level of the family increases the risk of obesity instead of decreasing it. This is probably due to the fact that well educated people are more likely to earn more money. We could say that even well educated people are not really aware of the burden of obesity for their lives.

The number of meals per day was a contributing factor. However, this result must be interpreted carefully, because dividing the total daily calories into small portions throughout the day is actually an effective means of controlling weight gain.(34, 35, 36)On the other hand, we think that in our subjects more frequent feeding probably increased the total amount of calories.

Obesity was more common in females and the prevalence increased with age. Higher education and older age of the parents was associated with higher income which is a risk factor for obesity in developing countries. Contrary to the trend in developed countries, obesity is more common in the affluent classes of the developing world.

Lack of correlation between TV hours and indices of obesity was in contrast to the results of other studies.(37, 38) However, the number of hours spent watching the TV, which averaged 2−3 hours a day, did not show significant individual variations in our study group.

In conclusion, we have found that the prevalence of childhood obesity is increasing in Turkey. Several risk factors have been identified that may provide target areas for community health care providers. Life−style changes have been widely accepted as the mainstay of obesity treatment. Two specific areas in our study population may be decreasing intake of beverages and reducing the use of the elevator. Prevention strategies must be developed and education to the community must be provided to increase public awareness and to implement healthy life styles.
